# Joint Iterative Decoding Design of Cooperative Downlink SCMA Systems

**DOI:** 10.3390/e27070762

**Published:** 2025-07-18

**Authors:** Hao Cheng, Min Zhang, Ruoyu Su

**Affiliations:** 1 School of Internet of Things, Nanjing University of Posts and Telecommunications, Nanjing 210003, China; haocheng@njupt.edu.cn (H.C.); minzhang@njupt.edu.cn (M.Z.); 2 National Mobile Communications Research Laboratory, Southeast University, Nanjing 210096, China

**Keywords:** SCMA, cooperative, multiuser detection, joint factor graph, closed form BER

## Abstract

Sparse code multiple access (SCMA) has been a competitive multiple access candidate for future communication networks due to its superiority in spectrum efficiency and providing massive connectivity. However, cell edge users may suffer from great performance degradations due to signal attenuation. Therefore, a cooperative downlink SCMA system is proposed to improve transmission reliability. To the best of our knowledge, multiuser detection is still an open issue for this cooperative downlink SCMA system. To this end, we propose a joint iterative decoding design of the cooperative downlink SCMA system by using the joint factor graph stemming from direct and relay transmission. The closed form bit-error rate (BER) performance of the cooperative downlink SCMA system is also derived. Simulation results verify that the proposed cooperative downlink SCMA system performs better than the non-cooperative one.

## 1. Introduction

Non-orthogonal multiple access (NOMA) [1,2,3] has been considered as one of the key technologies of beyond fifth-generation and sixth-generation communication networks [4,5] to provide high spectrum efficiency and massive connectivity, which are essential to Internet of Things (IoT) applications [6,7,8,9]. The principle of NOMA allows multiple users to occupy the same time and frequency resources. There are two kinds of NOMA techniques, i.e., the power domain NOMA (PD-NOMA) and the code domain NOMA [10,11,12]. In PD-NOMA, all the users are allocated with different levels of power according to the strength of channel state information from the base station (BS), and successive interference cancellation is performed at the receive end to apply multiuser detection. In code domain NOMA, such as sparse code multiple access (SCMA) [13,14,15,16], multiple user shared access, pattern division multiple access and interleave division multiple access [17,18], the loading factor is high and thus can provide large connectivity and high spectrum efficiency in comparison to orthogonal multiple access. In SCMA, the most popular code domain NOMA scheme, each user is equipped with a unique codebook, where several carefully designed codewords are involved. At the transmitter, the information bits are mapped to the multi-dimensional sparse codewords and then spread to orthogonal resources. The sparsity of the codewords enables the widely used message passing algorithm (MPA) detector to recover transmitted bits.

In recent years, most studies related to SCMA have focused on codebook design and low-complexity receivers. The authors in [19] propose a joint multiuser codebook design for uplink SCMA systems over Rayleigh fading channels based on the cutoff rate criterion of the equivalent multiple-input multiple-output system. By means of the golden angle modulation points, the authors in [20] provide two schemes for codebook design with a different number of optimization parameters, being independent of system configuration and simple to implement. An efficient SCMA codebook has been designed in [21] by introducing a one-dimensional searching algorithm to minimize the upper bound of pair-wise error probability. In [22], a novel codebook with large minimum Euclidean distance is provided by employing the star quadrature amplitude modulation signal constellations to meet the pair-wise error probability-based design criteria. The systematic construction procedure for the SCMA codebook design under various channel environments has been considered for small-scale application to achieve near-optimal performance, where the steps of rotation angle design, multi-dimensional codebook construction and labeling rule design are involved [23]. Unlike most existing codebook design structures, the authors in [24] present a novel class of an SCMA codebook design method, where power imbalance among different users is introduced instead of assuming uniform power allocation, by using the Star-QAM mother constellation and genetic algorithm in order to optimize the minimum Euclidean distance and the minimum product distance, respectively. Recent studies also focus on the SCMA codebook design with respect to specific channels. For the additive white Gaussian noise (AWGN) channel, a separable codebook scheme has been provided in [25] to simplify the maximization of the minimum Euclidean distance, where the multiple one-dimensional complex codebooks related to the resource element are maximized in terms of these criteria. By using the separable codebook structure to design heavily overloaded SCMA codebooks, the authors in [26] formulate a non-convex problem of minimizing codebook power under the distance constraints; then, the convex–concave procedure is adopted to solve it. By exploring the probability distribution of the Rician random variables and its indicators of multi-dimensional constellations, an enhanced codebook for various Rician scenarios has been generated in [27]. Based on the cross-entropy method, novel multiple-input multiple-output SCMA codebooks have been developed for both uplink and downlink Rayleigh fading channels [28].

In general, low-complexity decoders for SCMA can be classified into two categories: reducing the computation cost of the original MPA detector and exploring more efficient ones. The authors in [29] put forward a threshold-based MPA by setting a belief threshold to build an early stop criterion of the iteration process. The partial marginalization [30] MPA aims to reduce the computation complexity of the original one at the cost of negligible bit-error rate (BER) degradation by propagating messages the same as the original MPA role and determining part codewords at the *m*-th iteration. To accelerate its convergence, the authors in [31] provide the asynchronous MPA detector by allowing all the likelihood probabilities to update in a parallel manner. However, the ascending order of this strategy is not always the best choice; in this way, an improved serial scheduling-based MPA has been proposed in [32] to further accelerate its convergence, where the edges are selected according to the maximum value of the messages. The authors in [33] optimize the original MPA in two aspects: a lookup table is adopted to reduce the computational cost of the message exchanging instead of the extensive Jacobian approximation; thus, the stable convergence of the MPA can be guaranteed. The convergence of the original MPA is sped up by using a series of novel scheduling schemes. By extending stochastic computing to MPA [34], a low-complexity stochastic detector has been proposed by designing three novel stochastic logic architectures with low hardware cost bit-stream generation and resource node updating architecture, as well as the fast converging stochastic variable node updating rule. By using its lattice structure, a low-complexity decoding algorithm based on list sphere decoding [35], where the exhaustive search for all possible hypotheses, can be avoided. By narrowing down the range of believable superposed constellation points, a novel sphere decoding-based MPA structure is proposed in [36]. A low-complexity attempt for joint SCMA and generalized frequency-division multiplexing detection has been reported in [37] by virtue of the frequency-domain implementation, where the closed form average BER bound is also investigated. A novel joint iteration detection and decoding of multiple-input multiple-output and SCMA scheme has been investigated in [38] by designing the corresponding virtual sparse factor graph. An improved approximate message passing SCMA detection scheme has been provided in [39] for the imperfect channels in low-Earth-orbit satellite communications. The authors in [40] propose a linear minimum mean square error-based detection structure to construct an efficient transceiver design for affine frequency-division multiplexing-aided sparse code multiple access system. To address the inflexibility of user grouping and the diverse data rate support in SCMA, a variable modulation scheme has been proposed in [41] to allow different users to employ codebooks with diverse modulation orders. Recently, the artificial intelligence-based techniques in SCMA design and optimization have gained much attention in the literature. For example, to enable near-optimal codebook and low-complexity decoder design, an auto-encoder-based encoding–decoding structure for SCMA has been proposed in [42].

The cooperative transmission decode-and-forward strategy is able to enhance the performance of communication systems, especially for cell edge nodes, by using dedicated relays or allowing users to act as the relay in order to provide spatial diversity. This cooperative strategy has attracted much attention in PD-NOMA systems [43]. The cooperative NOMA scheme is first proposed in [43] by fully exploiting the prior knowledge. The authors in [43] also analyze the achieved outage probability and diversity order. The authors in [44] propose a two-stage relay selection strategy for cooperative NOMA by allowing users to require different qualities of service. The closed form expressions of the outage probabilities and diversity order for this cooperative NOMA are also determined. The closed form ergodic sum rate and outage probability of the downlink cooperative NOMA system have been studied over Nakagami-m fading channels under both decode-and-forward and amplify-and-forward protocols [45], where only statistical channel state information is available. The spatial modulation-aided cooperative NOMA [46] has been proposed for multiple-input and multiple-output systems, where the bit-error rate and ergodic sum-rate metrics have been investigated. A cooperative NOMA scheme is proposed to enhance the quality of user experience of the extended reality devices by striking a trade-off between the system throughput and fairness [47]. Bit-error rates, achievable rates and outage probabilities [48] are investigated for the two-stage massive multiple-input multiple-output cooperative relay NOMA network. The discrete-time Markov chain is utilized to demonstrate the processes of charging and discharging of the relay station under amplified-and-forward and decoded-and-forward cooperative transmission modes [49]. Then, the closed-form outage probabilities are also considered through mathematical analysis.

Apart from many solutions for cooperative PD-NOMA systems, this paradigm has just emerged for the cooperative SCMA system in [50], where exact expressions of average outage probabilities are analyzed. However, the efficient decoding strategy has not been considered. To this end, we propose the cooperative downlink SCMA system to enhance system performance, where the relay works in the decode-and-forward manner. Moreover, to utilize the received signal in two phases, the joint iterative decoding strategy is designed for this downlink cooperative SCMA system by stacking the signal from the direct link and the relay together. Closed-form BER performance is also derived. The main contributions of this paper are summarized as follows:

- The cooperative downlink SCMA system is introduced to enhance its performance robustness where two-phase transmission is involved, i.e., the direct link and the relay transmission.
- By stacking the signal from both the direct link and the relay transmission together, we propose a joint iterative decoding design of the cooperative downlink SCMA system with the aid of the augmented codebook and the joint virtual factor graph.
- To investigate the BER performance of cooperative downlink SCMA system, a closed-form solution is also provided.
- Simulation results confirm the validity of our proposed joint iterative decoding design of the cooperative downlink SCMA system, which can significantly improve the BER performance as compared to the non-cooperative one.

The rest of this paper is organized as follows. Section 2 introduces the system model for the downlink cooperative SCMA systems. In Section 3, we propose a joint iterative decoding scheme for the cooperative SCMA system detection. Section 4 provides the BER analysis for the cooperative SCMA system. Numerical results are provided in Section 5, followed by conclusions in Section 6.

## 2. System Model

This section briefly reviews the model of the downlink SCMA and introduces the cooperative downlink SCMA system.

### 2.1. SCMA System Description

**Figure 1:**
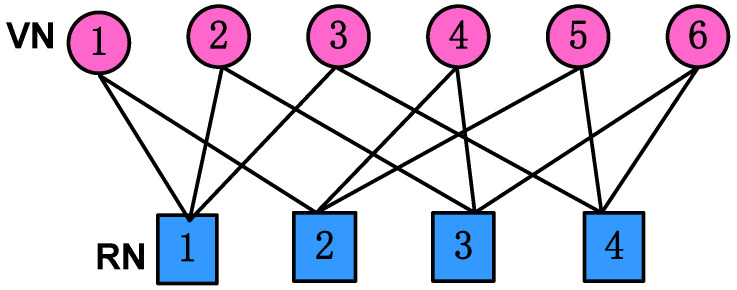
The SCMA factor graph for K=4 and J=6.

We consider the downlink SCMA system with *J* users spreading over *K* orthogonal resources served by a base station (BS). The overloading factor is defined as λ=J/K. Each user transmits its signal over $d_{v}^{\prime }$ orthogonal resources, while each orthogonal resource is occupied by $d_{c}^{\prime }$ users. This structure can be represented by a sparse binary K×J indicator matrix $F^{\prime }$, where the columns and rows denote the users and orthogonal resources, respectively. The element in the *k*-th row and the *j*-th column of the indicator matrix F′ is denoted as $f_{k,j}^{\prime }$, whose value is $f_{k,j}^{\prime }=1$ when the *k*-th resource node (RN) and the *j*-th variable node (VN) are connected; otherwise, $f_{k,j}^{\prime }=0$. In this work, the typical 4×6 and 5×10 indicator matrices are taken into consideration, given by

(1) $$\begin{array}{c} F_{4\times 6}^{\prime }=\left[\begin{array}{cccccc} 1 & 1 & 1 & 0 & 0 & 0\\ 1 & 0 & 0 & 1 & 1 & 0\\ 0 & 1 & 0 & 1 & 0 & 1\\ 0 & 0 & 1 & 0 & 1 & 1 \end{array}\right], \end{array}$$

and

(2) $$\begin{array}{c} F_{5\times 10}^{\prime }=\left[\begin{array}{cccccccccc} 1 & 1 & 1 & 1 & 0 & 0 & 0 & 0 & 0 & 0\\ 1 & 0 & 0 & 0 & 1 & 1 & 1 & 0 & 0 & 0\\ 0 & 1 & 0 & 0 & 1 & 0 & 0 & 1 & 1 & 0\\ 0 & 0 & 1 & 0 & 0 & 1 & 0 & 1 & 0 & 1\\ 0 & 0 & 0 & 1 & 0 & 0 & 1 & 0 & 1 & 1 \end{array}\right], \end{array}$$

where the loading factors are λ=150% and λ=200%, respectively. The column weights of both $F_{4\times 6}^{\prime }$ and $F_{5\times 10}^{\prime }$ are $d_{v}^{\prime }=2$, while the row weights of $F_{4\times 6}^{\prime }$ and $F_{5\times 10}^{\prime }$ are $d_{c}^{\prime }=3$ and $d_{c}^{\prime }=4$, respectively. The corresponding factor graph of the indicator matrix $F_{4\times 6}$ is depicted in Figure 1.

Based on the SCMA principle, each user is equipped with a unique complex codebook $C^{\prime }$, where *M* sparse codewords with *K*-dimensions are involved. This sparsity reduces the interference from other users and enables the message passing algorithm (MPA) to carry out detection.

### 2.2. The Cooperative SCMA System

**Figure 2:**
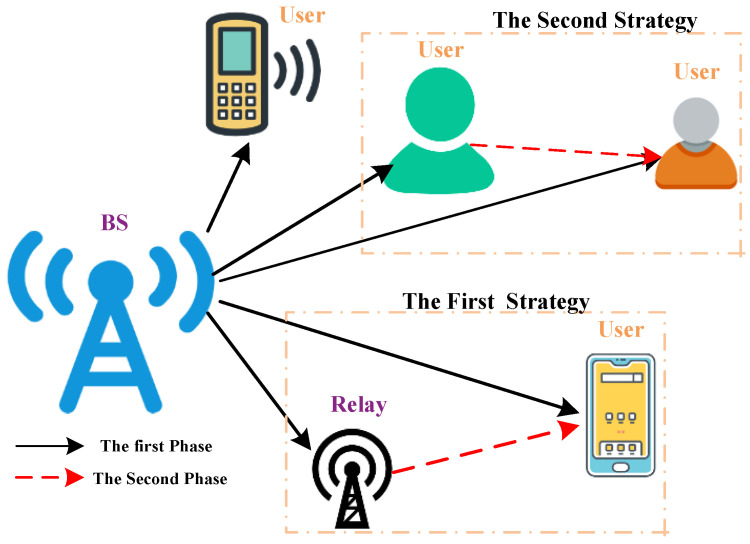
The system model of a cooperative downlink SCMA system.

As shown in Figure 2, the two-phase cooperative transmission strategy is deployed. We assume that these *J* users are partitioned into two groups, with the first group being located near the BS and the other group being far from the BS and needing a relay to improve system performance. The relay is assumed to work in a half-duplex manner, i.e., it cannot transmit and receive message simultaneously. Specially, this relay is used to decode-and-forward (DF) the message. In the first phase, the BS broadcasts the superimposed signal to all the users and the relay. In the second phase, the relay carries out SCMA detection and then broadcasts the detected messages after SCMA modulation.

**Remark** **1.** *The second strategy for the cooperative transmission is to allow the user close to the second group to act as the relay. Specially, in the first phase, the BS broadcasts the superimposed signal to all the users; in the second phase, the above-mentioned user carries out SCMA detection and then broadcasts the detected messages to the users in the second group after SCMA modulation. Since the joint iterative decoding design and the BER analysis of these two transmission strategies are the same, we focus on the first transmission strategy in this work.*

In what follows, we first formulate the received signal of the *j*-th user within the first group and the relay in the first phase, and then the received signal of the *j*-th user in the second group is introduced.

In the first phase, the received signal $y_{j}^{f}={\left[y_{1,j}^{f},y_{1,j}^{f},\cdots ,y_{K,j}^{f}\right]}^{T}$ of the *j*-th user can be represented as

(3) $$\begin{array}{c} y_{j}^{f}=\mathrm{diag}\left(h_{j}^{f}\right)\sum_{i=1}^{J}x_{i}^{b}+n_{j}^{f}, \end{array}$$

where $x_{i}^{b}={\left[x_{1,i}^{b},x_{2,i}^{b},\cdots ,x_{K,i}^{b}\right]}^{T}$ is the transmitted codeword of the *i*-th user, i=1,2,⋯,J, $h_{j}^{f}={\left[h_{1,j}^{f},h_{2,j}^{f},\ldots ,h_{K,j}^{f}\right]}^{T}$ denotes the channel gain vector between the BS and *j*-th user, $\mathrm{diag}(h_{j}^{f})$ is a diagonal matrix with its *k*-th element being $h_{k,j}^{f}$, and $n_{j}^{f}={\left[n_{1,j}^{f},n_{2,j}^{f},\ldots ,n_{k,j}^{f}\right]}^{T}$, in which $n_{k,j}^{f}$ is the zero-mean additive white Gaussian noise (AWGN) with variance $\sigma_{f}^{2}$. Similarly, the received signal $y_{r}={\left[y_{1,r},y_{1,r},\cdots ,y_{K,r}\right]}^{T}$ of the relay can be written as

(4) $$\begin{array}{c} y_{r}=\mathrm{diag}\left(h_{r}\right)\sum_{i=1}^{J}x_{i}^{b}+n_{r}, \end{array}$$

where $h_{r}={\left[h_{1,r},h_{2,r},\ldots ,h_{K,r}\right]}^{T}$ is the channel gain vector between the BS and relay; $n_{r}={\left[n_{1,r},n_{2,r},\ldots ,n_{k,r}\right]}^{T}$ is also modeled as the zero mean AWGN with covariance $E\left[n_{r}n_{r}^{H}\right]=\sigma_{r}^{2}I_{K}$.

In the second phase, the relay decodes the transmitted signal of all the users, i.e., $x_{j}^{r}={\left[x_{1,j}^{r},x_{2,j}^{r},\cdots ,x_{K,j}^{r}\right]}^{T}$, and broadcasts the superimposed signal which is far from the BS to these users in the group. In this way, the received signal $y_{j}^{s}={\left[y_{1,j}^{s},y_{1,j}^{s},\cdots ,y_{K,j}^{s}\right]}^{T}$ of the *j*-th user in the second group can be denoted as

(5) $$\begin{array}{c} y_{j}^{s}=\mathrm{diag}\left(h_{j}^{s}\right)\sum_{i=1}^{J}x_{i}^{r}+n_{j}^{s}, \end{array}$$

where $h_{j}^{s}={\left[h_{1,j}^{s},h_{2,j}^{s},\ldots ,h_{K,j}^{s}\right]}^{T}$ is the channel gain vector between the relay and user *j* in the second group, $n_{j}^{s}={\left[n_{1,j}^{s},n_{2,j}^{s},\ldots ,n_{k,j}^{s}\right]}^{T}$ is the zero-mean AWGN with covariance $\sigma_{s}^{2}I_{K}$. To simplify the representation, the received signal of the first and second phase for the *i*-th user in the second group can be rewritten as

(6) $$\begin{array}{c} y^{f}=\mathrm{diag}\left(h^{f}\right)\sum_{i=1}^{J}x_{i}^{b}+n^{f},y^{s}=\mathrm{diag}\left(h^{s}\right)\sum_{i=1}^{J}x_{i}^{r}+n^{s}, \end{array}$$

where the subscript “j” is removed and $y^{a}={\left[y_{1}^{a},y_{1}^{a},\cdots ,y_{K}^{a}\right]}^{T}$, $h^{a}={\left[h_{1}^{a},h_{2}^{a},\ldots ,h_{K}^{a}\right]}^{T}$, $n^{a}={\left[n_{1}^{a},n_{2}^{a},\ldots ,n_{k}^{a}\right]}^{T}$, a=f,s.

## 3. Joint Iterative Decoding Design of a Cooperative SCMA System

In this section, we propose the joint iterative decoding design of a cooperative SCMA system. Specially, we first reconstruct the indicator matrix for the users within the second group by integrating the received signal in the first and second phases, and then the iterative decoding process is introduced with the help of a joint factor graph.

After the data transmission in two phases, both the signal of the direct transmission and the relay are received. With $y^{f}$ and $y^{s}$ in hand, by stacking the signal from the direct link and the relay together, the received signal of the *j*-th user in the second group can be described as

(7) $$\begin{array}{c} y=\mathrm{diag}\left(h\right)\sum_{j=1}^{J}x_{j}+n, \end{array}$$

where

(8) $$\begin{array}{c} y=\left[\begin{array}{c} y^{f}\\ y^{s} \end{array}\right]=\left[\begin{array}{c} y_{1}^{f}\\ \vdots \\ y_{K}^{f}\\ y_{1}^{s}\\ \vdots \\ y_{K}^{s} \end{array}\right]=\left[\begin{array}{c} y_{1}\\ \vdots \\ y_{2K} \end{array}\right],h=\left[\begin{array}{c} h^{f}\\ h^{s} \end{array}\right]=\left[\begin{array}{c} h_{1}^{f}\\ \vdots \\ h_{K}^{f}\\ h_{1}^{s}\\ \vdots \\ h_{K}^{s} \end{array}\right]=\left[\begin{array}{c} h_{1}\\ \vdots \\ h_{2K} \end{array}\right], \end{array}$$

and

(9) $$\begin{array}{c} x_{j}=\left[\begin{array}{c} x_{j}^{b}\\ x_{j}^{r} \end{array}\right]=\left[\begin{array}{c} x_{1,j}^{b}\\ \vdots \\ x_{K,j}^{b}\\ x_{1,j}^{r}\\ \vdots \\ x_{K,j}^{r} \end{array}\right]=\left[\begin{array}{c} x_{1,j}\\ \vdots \\ x_{2K,j} \end{array}\right],n=\left[\begin{array}{c} n^{f}\\ n^{s} \end{array}\right]=\left[\begin{array}{c} n_{1}^{f}\\ \vdots \\ n_{K}^{f}\\ n_{1}^{s}\\ \vdots \\ n_{K}^{s} \end{array}\right]=\left[\begin{array}{c} n_{1}\\ \vdots \\ n_{2K} \end{array}\right]. \end{array}$$

As can be seen in (7), the sparse feature of this downlink cooperative SCMA system can be represented by a binary 2K×J virtual indicator matrix F, whose element at the *u*-th row and the *j*-th column is denoted as $f_{u,j},u=1,2,\cdots ,2K$, given by

(10) $$\begin{array}{c} F=\left[\begin{array}{c} F^{\prime }\\ F^{\prime } \end{array}\right]. \end{array}$$

It is clear that the column weight of F is two times that of $F^{\prime }$, while the row weight of F is identical to that of $F^{\prime }$, giving us

(11) $$\begin{array}{c} d_{c}=d_{c}^{\prime },d_{v}=2d_{v}^{\prime }. \end{array}$$

**Figure 3:**
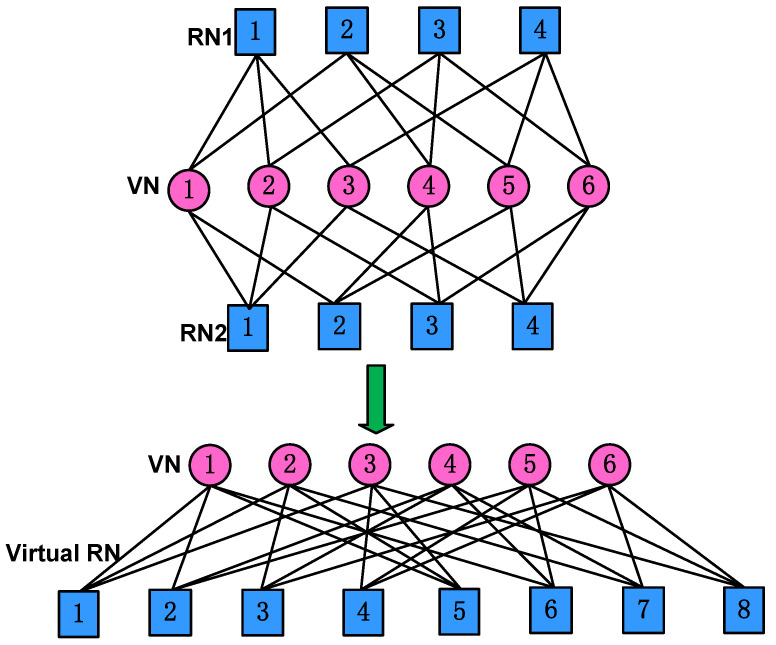
The virtual factor graph of 2K=8 and J=6 for the downlink cooperative SCMA system.

Intuitively, all the possible codewords of $x_{j}$ constitute the virtual codebook of the downlink cooperative SCMA system, given by

(12) $$\begin{array}{c} C=\left[\begin{array}{c} C^{b}\\ C^{r} \end{array}\right], \end{array}$$

where $C^{b}$ and $C^{r}$ are the codebooks of the transmitted signals $x_{j}^{b}$ and $x_{j}^{r}$, respectively. The virtual indicator matrix F can be represented by a joint factor graph, as shown in Figure 3, which consists of 2K=8 virtual resource nodes and J=6 variable nodes.

The near-optimal MPA detector can be applied to detect the transmitted bits for the downlink cooperative SCMA system, whose decoding process can be described as the exchanging of the marginal probability distribution of each virtual codeword between virtual RNs and VNs. The conventional probability domain MPA involves a great amount of exponentiation computation, which limits its application. In this way, the logarithm domain MPA is adopted. Before introducing the MPA, we define two sets

(13) $$\begin{array}{c} R_{j}=\left\{k|f_{k,j}=1\right\},j=1,2,\cdots ,J, \end{array}$$

and

(14) $$\begin{array}{c} V_{k}=\left\{j|f_{k,j}=1\right\},k=1,2,\cdots ,2K, \end{array}$$

to represent the set of virtual resource indices allocated to the *j*-th VN and the set of VNs sharing the *k*-th virtual RN. Note that the cardinality of $R_{j}$ and $V_{k}$ are $d_{v}$ and $d_{c}$, respectively. We also define $V_{k\to j}^{t}$ and $R_{j\to k}^{t}$ as the probability distributions propagating to the *k*-th virtual RN from the *j*-th VN and the *j*-th VN from the *k*-th virtual RN at the *t*-th iteration, respectively. The updating steps of the standard logarithm domain MPA can be described as follows:

(1) *Initialization*: Set the maximum iteration number as *T* and $R_{j\to k}^{0}=\log1/M$, i.e., the priori log-likelihood probability of each transmitted symbol is identical. Calculate the logarithm domain conditional probability as

(15) $$\begin{array}{c} P_{k}\left(x\right)=-\frac{1}{2\sigma^{2}}{\left|y_{k}-h_{k}\sum_{m\in V_{k}}x_{k,m}\right|}^{2},k=1,2,\cdots ,2K, \end{array}$$

where $x=\left\{x_{m}\right\},m\in V_{k}$.

(2) *Resource Node Updating*: At the *t*-th iteration, the message sent from the *k*-th virtual RN to the *j*-th VN is given by

(16) $$\begin{array}{c} V_{k\to j}^{t}\left(x_{j}\right)=\max_{x_{u}:u\in V_{k}\setminus j}^{\;\;\;\;\;\;\;\;*}\left\{P_{k}\left(x\right)+\sum_{u\in V_{k}\setminus j}R_{u\to k}^{t-1}\left(x_{u}\right)\right\},k=1,2,\cdots ,2K, \end{array}$$

where $V_{k}\setminus j$ denotes the $d_{c}-1$ VNs connected to the *k*-th virtual RN with the *j*-th VN excluded, and the operation $\max^{*}\left(.\right)$ is given by

(17) $$\begin{array}{c} \max^{\;\;\;\;\;\;\;\;*}\left(a_{1},a_{2},\cdots ,a_{n}\right)=\ln\left(e^{a_{1}}+e^{a_{2}}+\cdots +e^{a_{n}}\right). \end{array}$$

The computational cost of MPA detection mainly lies on the $\max^{*}\left(.\right)$ exponential operation above. To this end, Jacobian approximation is often used to reduce the computation cost of this calculation, giving us

(18) $$\begin{array}{c} \max^{\;\;\;\;\;\;\;\;*}\left\{a_{1},a_{2},\cdots ,a_{n}\right\}\approx \max\left\{a_{1},a_{2},\cdots ,a_{n}\right\}. \end{array}$$

In this way, the virtual resource node updating in (16) can be simplified into

(19) $$\begin{array}{c} V_{k\to j}^{t}\left(x_{j}\right)\approx \max_{x_{u}:u\in V_{k}\setminus j}\left\{P_{k}\left(x\right)+\sum_{u\in V_{k}\setminus j}R_{u\to k}^{t-1}\left(x_{u}\right)\right\},k=1,2,\cdots ,2K, \end{array}$$

(3) *Variable Node Updating*: At the *t*-th iteration, the message sent from the *j*-th VN to the *k*-th virtual RN can be computed as

(20) $$\begin{array}{c} R_{j\to k}^{t}\left(x_{j}\right)=\sum_{m\in R_{j}\setminus k}V_{m\to j}^{t-1}\left(x_{j}\right),k=1,2,\cdots ,2K, \end{array}$$

where $R_{j}\setminus k$ represents the $d_{v}-1$ RNs connected to the *k*-th VN with the *j*-th virtual RN excluded.

(4) *Probability Calculating and Symbol Judging*: When the algorithm is converged or the maximum number of iterations *T* is reached, a posteriori probability of virtual codeword $x_{j}$ can be calculated as

(21) $$\begin{array}{c} R_{j}\left(x_{j}\right)=\sum_{m\in R_{j}}V_{m\to j}^{T}\left(x_{j}\right). \end{array}$$

Note that the transmit virtual codeword ${\hat{x}}_{j}$ of user *j* in the second group is the one maximizing $R_{j}\left(x_{j}\right)$.

## 4. Complexity Analysis

In this section, the computational cost of the proposed joint iterative decoding MPA detector of the cooperative SCMA system is analyzed. The computational cost is measured in terms of the number of real multiplications, additions and comparisons involved in the iterations. Note that each complex multiplication consists of four real multiplications and two additions, while each complex addition consists of two real additions. In this sense, the total number of real multiplications is $4Kd_{c}+JMd_{v}^{2}$ in the direct transmission, while it becomes $8Kd_{c}+4JMd_{v}^{2}$ in the joint detection. The total number of additions and comparisons is 7K and $Md_{c}K\left(M^{d_{c}-1}-1\right)+MJ-J$ for the direct transmission and 14K and $2Md_{c}K\left(M^{d_{c}-1}-1\right)+MJ-J$ in the joint detection. This difference stems from the fact that the number of virtual resource nodes for joint detection is two times that of the direct transmission, or equivalently, the column weight of the indicator matrix F is two times that of $F^{\prime }$.

## 5. BER Performance Analysis of the Cooperative SCMA System

In this section, we investigate the BER performance of the proposed downlink cooperative SCMA system with perfect channel state information by assuming ideal cooperation, i.e., the relay detects the transmit codewords without errors, under maximum likelihood (ML) multiuser detection.

By defining $X=[x_{1},x_{2},\cdots ,x_{J}]$, according to the ML detection rule, the conditional pair-wise error probability that X is transmitted while $\hat{X}=\left[{\hat{x}}_{1},{\hat{x}}_{2},\cdots ,{\hat{x}}_{J}\right]$ is detected can be computed as

(22) $$\begin{array}{ccc} P_{s}\left\{X\to \hat{X}|h\right\}\;\;\; & = & \;\;\;Q\left(\sqrt{\frac{1}{2\sigma^{2}}{∥\mathrm{diag}\left(h\right)\left(X-\hat{X}\right)∥}^{2}}\right), \end{array}$$

where

(23) $$\begin{array}{c} Q\left(x\right)=\frac{1}{\sqrt{2\pi }}\int_{x}^{\infty }e^{-\frac{t^{2}}{2}}dt, \end{array}$$

is the Gaussian-Q function, which can be approximated as

(24) $$\begin{array}{c} Q\left(x\right)\approx \frac{1}{12}e^{-\frac{1}{2}x^{2}}+\frac{1}{6}e^{-\frac{2}{3}x^{2}}, \end{array}$$

and X consists of $M^{J}$ possible combined cases. Note that $\sigma^{2}$ is the average variance of the noise component of vector n in (9). The noise vector n in (9) consists of two parts, i.e., the noise from the direct transmission $n_{f}$ and the other from the relay transmission $n_{s}$; in this way, we define $\sigma^{2}$ as the average of this two parts, given by

(25) $$\begin{array}{c} \sigma^{2}=\frac{\sigma_{f}^{2}+\sigma_{s}^{2}}{2}, \end{array}$$

From the discussions above, the pair-wise error probability in (22) can be simplified into

(26) $$\begin{array}{c} P_{s}\left\{X\to \hat{X}|h\right\}=\frac{1}{12}e^{-\frac{∥\mathrm{diag}\left(h\right)\left(X-\hat{X}\right){∥}^{2}}{2(\sigma_{f}^{2}+\sigma_{s}^{2})}}+\frac{1}{6}e^{-\frac{2∥\mathrm{diag}\left(h\right)\left(X-\hat{X}\right){∥}^{2}}{3(\sigma_{f}^{2}+\sigma_{s}^{2})}} \end{array}$$

By defining $d(X,\hat{X})$ as the number of bits in which X differs from $\hat{X}$, the BER upper bound of the downlink cooperative SCMA system can be described as

(27) $$\begin{array}{c} P\leq \frac{1}{M^{J}}\sum_{X}\sum_{X\neq \hat{X}}d\left(X,\hat{X}\right)P_{s}\left\{X\to \hat{X}|h\right\}. \end{array}$$

## 6. Simulation Results

In this section, numerical simulation results were conducted to evaluate the BER performance of the proposed cooperative downlink SCMA system under the additive white Gaussian noise (AWGN) and Rayleigh channels. The SCMA system configurations in Equations (1) and (2) were considered, where the loading factors are λ=150% and λ=200%, respectively. The channel gain h was modeled as a independently and identically distributed circularly symmetric complex-valued Gaussian random variable with zero mean and covariance $\sigma^{2}I$. The codebook in [30] was adopted in the base station and relay.

### 6.1. BER Evolution for AWGN Channel

**Figure 4:**
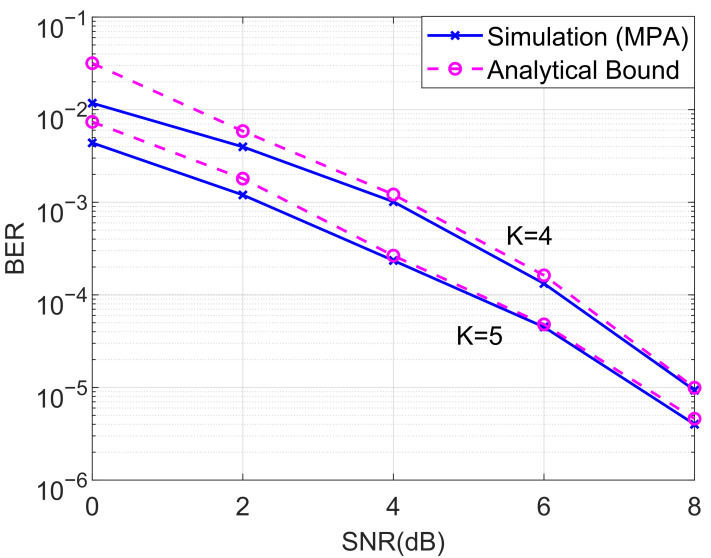
Comparison of the BERs of the analyzed and MPA detector for the proposed cooperative SCMA system under an AWGN channel with K=4 and K=5.

In this subsection, we investigated the BERs of the proposed downlink cooperative SCMA system under an AWGN channel. We first compared the BER performance of the analyzed and MPA detector for the proposed cooperative SCMA system with K=4 and K=5, as shown in Figure 4. The theoretical average BER was computed according to (27) and the steps of the MPA were discussed in Section 3. As can be seen from Figure 4, it is straightforward that the analytical bounds are almost overlapped with the simulated BERs for both K=4 and K=5 in the moderate and high-SNR regions.

**Figure 5:**
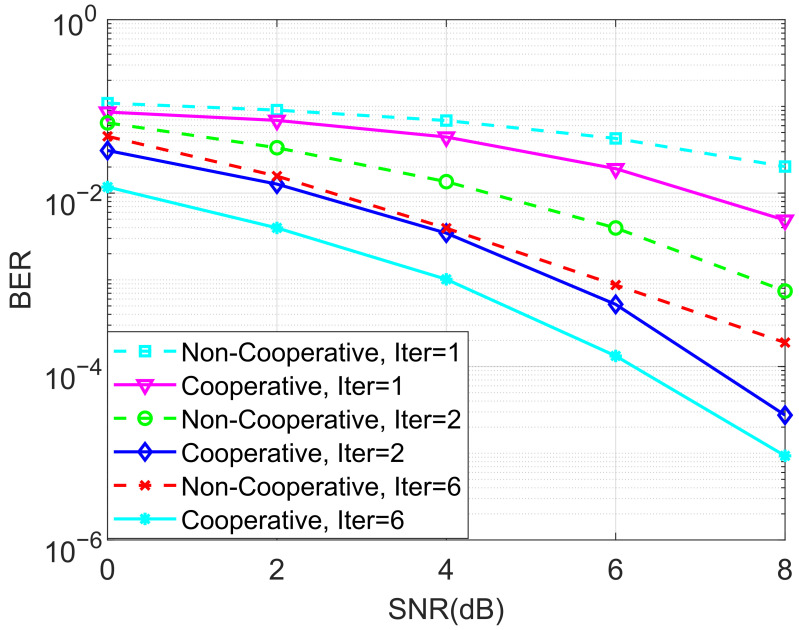
The BER performance of the proposed ideal cooperative SCMA system under an AWGN channel and different iterations of the MPA detector for K=4.

**Figure 6:**
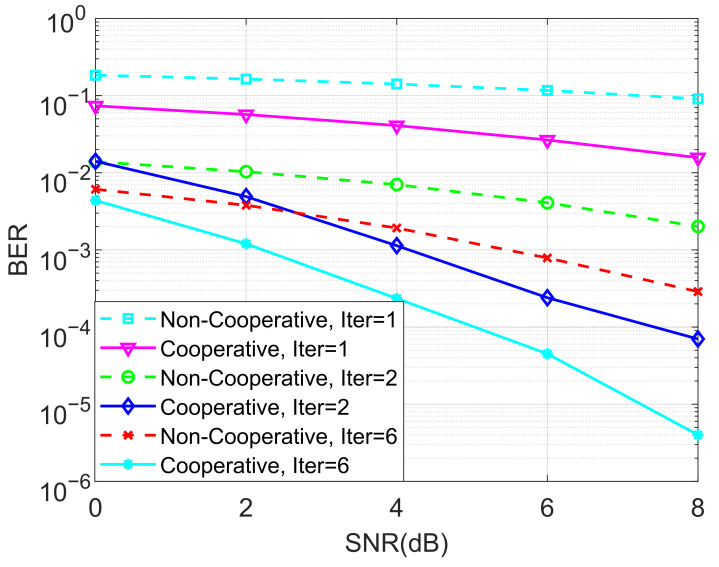
The BER performance of the proposed ideal cooperative SCMA system under an AWGN channel and different iterations of the MPA detector for K=5.

In the next stage of simulations, the BERs of the proposed ideal cooperative SCMA system under an AWGN channel and different iterations of the MPA detector for K=4 and K=5 are depicted in Figure 5 and Figure 6, respectively. It is observed that the BER performance of the cooperative SCMA system is better than that of the non-cooperative one. This is because the location of users in the second group is far from the BS, and the strength of the signal received by these users is relatively weak. Moreover, the cooperative SCMA system suffers from large decreases in BER performance when the number of iterations for the MPA detector is not large enough. Simulations demonstrated that six iterations can be used to arrive at the intended results.

**Figure 7:**
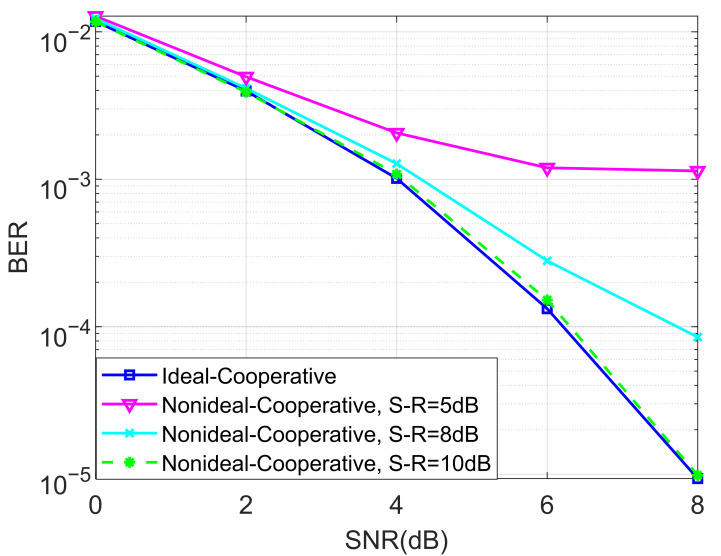
The BER performance of the proposed non-ideal cooperative SCMA system under an AWGN channel and different SNRs of the S-R link for K=4.

**Figure 8:**
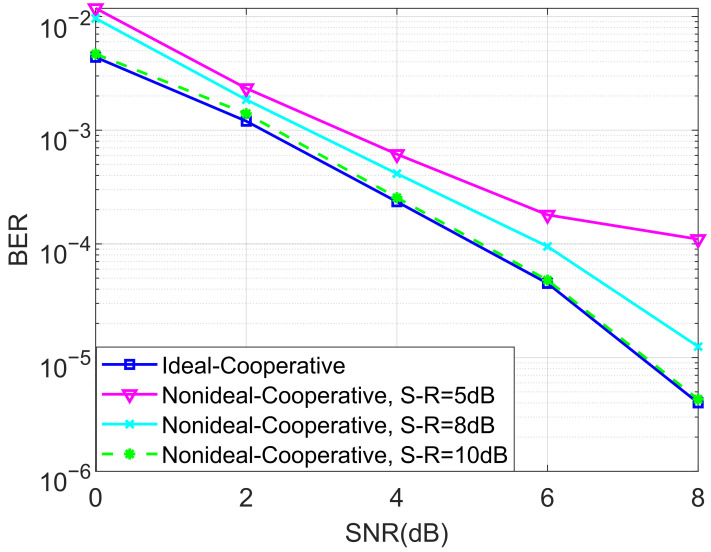
The BER performance of the proposed non-ideal cooperative SCMA system under an AWGN channel and different SNRs of the S-R link for K=5.

We also evaluated the BER performance of the proposed non-ideal cooperative SCMA system under an AWGN channel and different SNRs of the source–relay (S-R) link for K=4 and K=5 in Figure 7 and Figure 8, respectively. This is practical since the decoding of the SCMA codewords in the relay is often imperfect, i.e., the decoded bits suffer from a certain amount of errors. We claim that there is no detection error when the SNR of the S-R link is high. Observe from both Figure 7 and Figure 8 that the destination user achieves better BERs when the SNR of the S-R link becomes higher, which in return provides us with guidance for how to approach the design of the relay’s location.

### 6.2. BER Evolution for Rayleigh Channel

**Figure 9:**
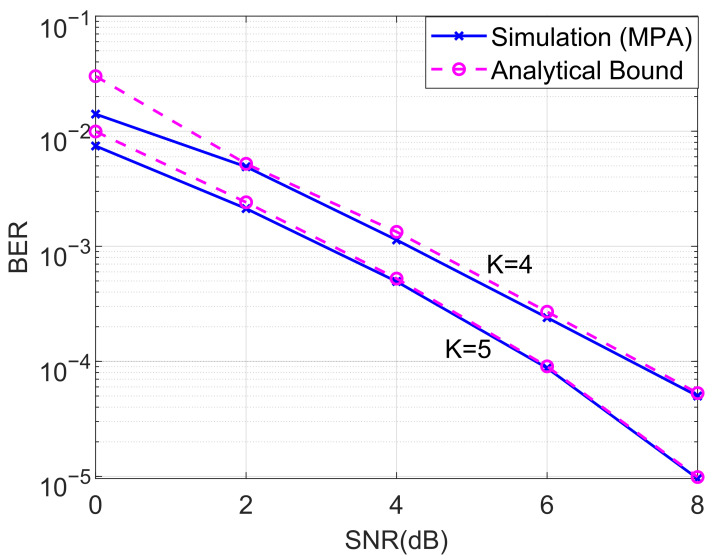
Comparison of the BERs and MPA detector for the proposed cooperative SCMA system under a Rayleigh channel with K=4 and K=5.

In this subsection, we verify the effectiveness of the proposed downlink cooperative SCMA system for the Rayleigh channel. We compared the BERs and MPA detector for the proposed cooperative SCMA system under a Rayleigh channel with K=4 and K=5 in Figure 9. All the cases verify the validity of our analysis results in (27). We assume an ideal cooperative case in the simulation. Actually, this ideal cooperative system can be achieved when the SNR of the S-R link is above a certain value.

**Figure 10:**
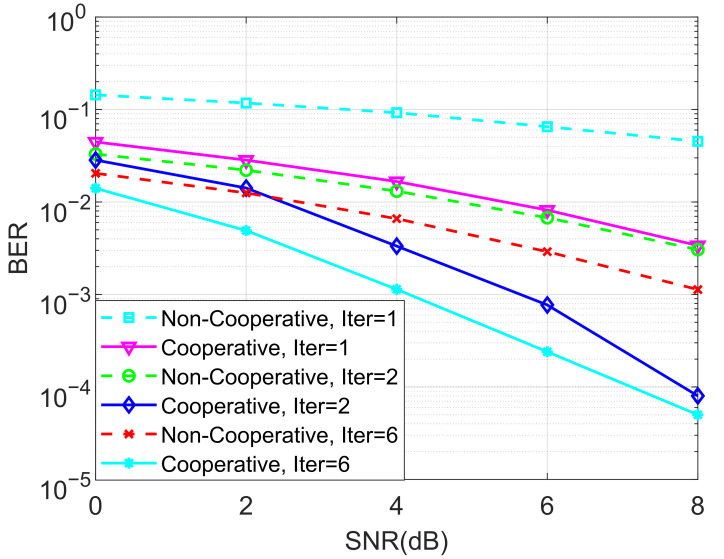
The BER performance of the proposed ideal cooperative SCMA system under a Rayleigh channel and different iterations of the MPA detector for K=4.

**Figure 11:**
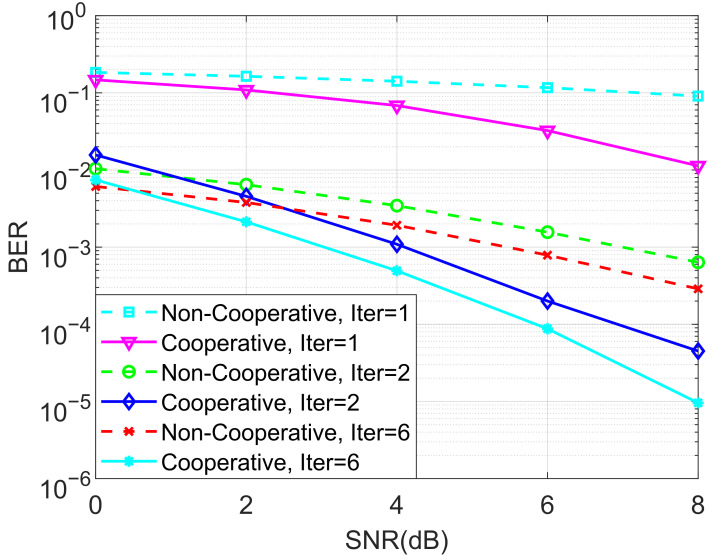
The BER performance of the proposed ideal cooperative SCMA system under a Rayleigh channel and different iterations of the MPA detector for K=5.

**Figure 12:**
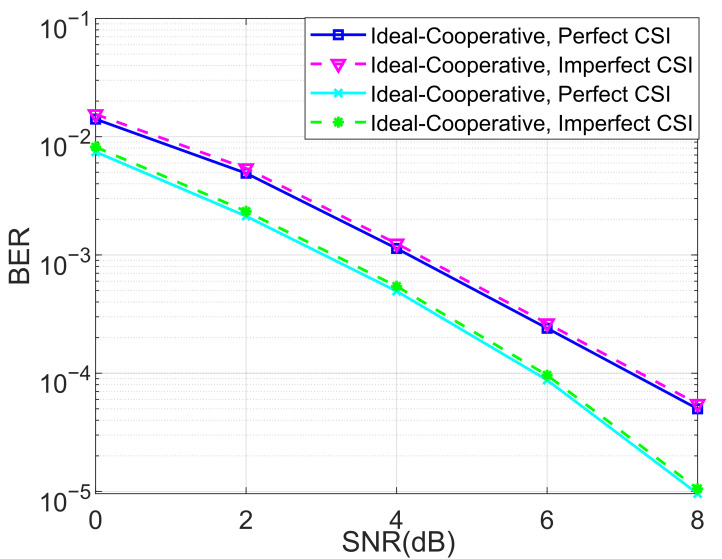
The BER performance of the proposed ideal cooperative SCMA system under a Rayleigh channel with imperfect channel state information for K=4 and K=5.

We also analyzed the BERs of the proposed ideal cooperative SCMA system under a Rayleigh channel with different numbers of iterations for the MPA detector under K=4 and K=5 in Figure 10 and Figure 11, respectively. The advantage of the cooperative SCMA system over the non-cooperative one was highlighted, demonstrating the validity of the designed cooperative system. By using the sparse-based channel estimator in [51], we also investigate the BER performance of the proposed ideal cooperative SCMA system under a Rayleigh channel with imperfect channel state information (CSI) for K=4 and K=5 in Figure 12. It is obvious that BER degradation is negligible, which further clarifies the robustness of the proposed joint detection structure.

**Figure 13:**
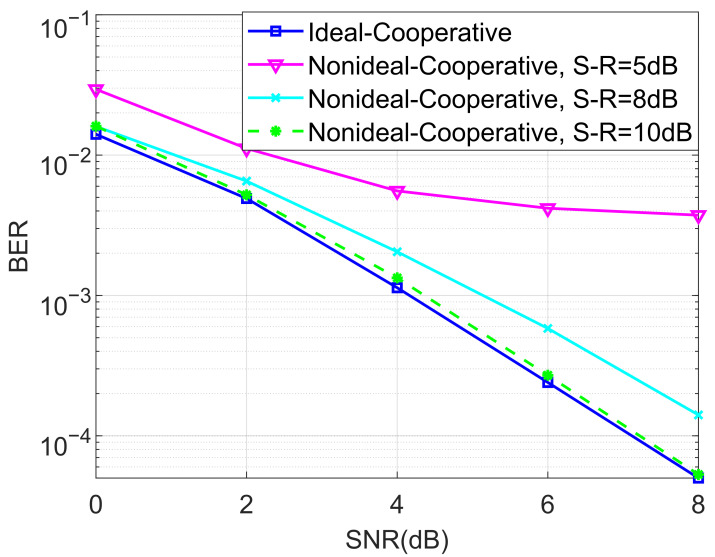
The BER performance of the proposed non-ideal cooperative SCMA system under a Rayleigh channel and different SNR of the S-R link for K=4.

**Figure 14:**
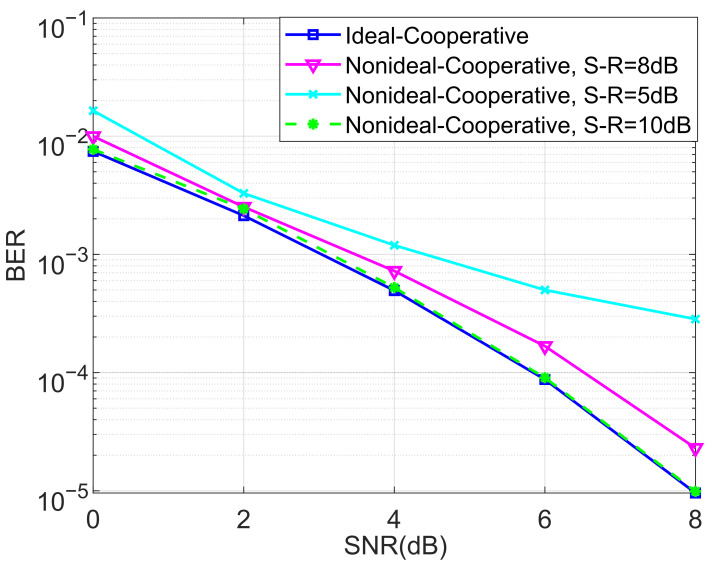
The BER performance of the proposed non-ideal cooperative SCMA system under a Rayleigh channel and different SNRs of the S-R link for K=5.

In the last set of simulations, the BER performance of the proposed non-ideal cooperative SCMA system over a Rayleigh channel and different SNRs of the S-R link for K=4 and K=5 were considered, as shown in Figure 13 and Figure 14, respectively. It can be observed from Figure 13 and Figure 14 that the non-ideal cooperative SCMA system’s performance decreases significantly when the SNR of the S-R link is only 5 dB. This is because the detected bits in the relay consist of a large amount of errors, propagating into the destination users, and a large SNR is more suitable for practical implementation.

## 7. Conclusions

This work provides a cooperative downlink SCMA system by using a relay to serve the cell edge users, which works in a decode-and-forward manner. To achieve better BER performance, we propose a joint iterative decoding design for the cooperative downlink SCMA system by means of a joint factor graph, which extends the original indicator matrix to the cooperative one. Moreover, the closed-form BER performance is also derived to enable us to perform analyses on the joint iterative MPA detector. Simulation results demonstrate the superiority of the downlink cooperative SCMA system over the non-cooperative one.

## Data Availability

Data are contained within this article.
